# Psychometric assessment of oral health-related quality of life questionnaires cross-culturally adapted for use in Brazilian adults - a systematic review

**DOI:** 10.1590/1980-549720230046

**Published:** 2023-10-27

**Authors:** Yure Gonçalves Gusmão, José Cristiano Ramos Glória, Maria Letícia Ramos-Jorge, Frederico Santos Lages, Dhelfeson Willya Douglas-de-Oliveira

**Affiliations:** I Universidade Federal dos Vales do Jequitinhonha e Mucuri - Diamantina (MG), Brazil.; II Universidade Federal de Minas Gerais - Belo Horizonte (MG), Brazil.

**Keywords:** Quality of life, Questionnaires, Validation study, Psychometrics, Systematic review, Qualidade de vida, Questionários, Estudo de validação, Psicometria, Revisão sistemática

## Abstract

**Objective:**

This study aimed to review the psychometric properties of oral health-related quality of life (OHRQoL) questionnaires for the Brazilian adult population.

**Methods:**

A systematic review was performed based on the COSMIN guidelines (PROSPERO CRD42022300018). The studies were obtained through electronic searches in the PubMed/MEDLINE, Web of Science, Lilacs, VHL (BIREME), SciELO, and Embase databases.

**Results:**

The search was performed in December 2022. Articles on OHRQoL that reported the cross-cultural adaptation of instruments into Portuguese (Brazil) and evaluated the psychometric properties of measuring instruments in adult patients were included. Those about the development of a novel instrument and participants under 18 years of age were excluded. Information was collected on the country, type of instrument validated, psychometric tests, and adaptation process. The certainty of the evidence was assessed using GRADEpro program. The search returned 6,556 articles, and 14 were considered for this review. However, two studies did not report the cross-cultural adaptation process. Content validity, internal consistency, criterion validity, construct validity, reliability, general discriminant validity, Cronbach’s alpha value, and general intraclass correlation coefficient value were confirmed in 12 studies. Cronbach’s alpha ranged from 0.69 to 0.96. The certainty of the evidence was considered moderate and low. This study has some limitations, such as the lack of information in some reviewed studies, the unavailability of Brazilian instruments, and absence of longitudinal validation of some instruments.

**Conclusions:**

In conclusion, there are 14 OHRQoL instruments adapted for Brazilian adults that can be used with caution by researchers and clinicians, since they presented moderate to low certainty of the evidence.

## RESUMO

**Objetivo:**

Este estudo teve como objetivo revisar as propriedades psicométricas dos questionários de qualidade de vida relacionada à saúde bucal (OHRQoL) para a população adulta brasileira.

**Métodos:**

Foi realizada uma revisão sistemática com base nas diretrizes *Consensus-based standards for the selection of health measurement instruments* - COSMIN (*International Prospective Register of Systematic Reviews* - PROSPERO CRD42022300018). Os estudos foram obtidos por meio de buscas eletrônicas nas bases de dados *United States National Library of Medicine* (PubMed)/ *Medical Literature Analysis and Retrieval System Online* (MEDLINE), *Web of Science*, Literatura Latino-Americana e do Caribe em Ciências da Saúde (Lilacs), Biblioteca Virtual em Saúde - BVS (Centro Latino-Americano e do Caribe de Informação em Ciências da Saúde - BIREME), *Scientific Electronic Library Online* (SciELO) e Embase. A busca foi realizada em dezembro de 2022. Foram incluídos artigos que relatavam a adaptação transcultural de instrumentos (QVRSB) para o português (Brasil) e que avaliavam as propriedades psicométricas de mensuração de instrumentos (QVRSB) em pacientes adultos. Foram excluídos aqueles sobre o desenvolvimento de um novo instrumento e com participantes menores de 18 anos. Foram coletadas informações sobre país, tipo de instrumento validado, testes psicométricos e processo de adaptação. A certeza da evidência foi avaliada usando GRADE.

**Resultados:**

A pesquisa retornou 6556 artigos, e 14 foram incluídos nesta revisão. Dois estudos não relataram o processo de adaptação transcultural. A validade de conteúdo, consistência interna, validade de critério, validade de constructo, confiabilidade, validade discriminante geral, valor alfa de Cronbach e valor geral do coeficiente de correlação intraclasse foram confirmadas em 12 estudos. O alfa de Cronbach variou de 0,69 a 0,96. A certeza da evidência foi considerada moderada e baixa. Esta pesquisa apresenta algumas limitações, como falta de informação em alguns estudos revisados; indisponibilidade de instrumentos brasileiros; ausência de validação longitudinal de alguns instrumentos.

**Conclusões:**

Em conclusão, existem 14 instrumentos de QVRSB adaptados para adultos brasileiros que podem ser utilizados com cautela por pesquisadores e clínicos, uma vez que apresentam moderada a baixa certeza de evidência.

## INTRODUCTION

Quality of life is defined as the individuals’ perception of their position in life in the context of the culture and value systems in which they are inserted, as well as their goals, expectations, standards, and concerns^1^ ^,^ ^2^. The part concerning quality of life affected by oral health and orofacial conditions is named Oral Health-Related Quality of Life (OHRQoL). It investigates how oral health can affect function, psychological state, social factors, and pain or discomfort of individuals^3^.

To understand the aspects covered by the OHRQoL, a multidimensional evaluation based on specific or generic structured questionnaires is used. This can offer the researchers a wide selection of options to use different instruments according to the objectives of their research, in addition to allowing the evaluation of the impacts of the orofacial health condition in an individual’s life through the psychological, physical, and social dimensions^4^. However, these questionnaires have limitations in their applicability when they are developed in English-speaking countries and with sociocultural realities different from Brazil. In these cases, the questionnaire must undergo a process of cross-cultural adaptation and psychometric validation before being used in Brazil^5^.

The equivalence and preservation of these instruments are outlined by standardized validation and cross-cultural adaptation guidelines, consisting of six stages:

1. Translation;
2. Synthesis;
3. Reverse translation;
4. Review by the expert committee;
5. Test of the pre-final version, and
6. Submission and evaluation of all reports written by the committee^6^.

In addition, these instruments must ensure the reliability of their results through psychometric properties that use quality criteria for their measurements, such as content validity, internal consistency, construct validity, reproducibility, responsiveness, convergent validity, discriminant validity, and interpretation^5^ ^,^ ^7^.

Some OHRQoL questionnaires have been translated into Brazilian Portuguese and are available for use^8^ ^,^ ^9^. However, there is a gap between the reliability and the cross-cultural adaptation method employed, requiring a critical evaluation of these translated versions to verify the adapted measure and the preservation of the original instrument’s psychometric properties.

This systematic review aimed to reassess the psychometric properties of OHRQoL questionnaires adapted for the Brazilian adult population and identify their suitability for research and clinical practice in Brazil.

## METHODS

The present systematic review is registered in the International Prospective Register of Systematic Reviews (PROSPERO) under CRD42022300018, and was performed based on the Consensus-based Standards for the Selection of Health Measurement Instruments (COSMIN) guidelines for systematic reviews of patient-reported outcome measures (PROMs)^10^. The outcomes of interest were psychometrics and cross-cultural adaptation.

### PICO question

Are oral health-related quality of life instruments cross-culturally adapted for application in Brazilian adults reliable?

### Eligibility criteria

For this systematic review, studies that met the following inclusion criteria were selected:

1. Validation and cross-cultural adaptation studies of OHRQoL instruments into Brazilian Portuguese;
2. Studies that evaluated the psychometric properties of measurement of OHRQoL instruments in adult patients; and
3. Studies that reported at least one of the measurement properties: reliability, internal consistency, measurement error, content validity, construct validity, criterion validity, discriminant validity, and/or convergent validity.

Systematic reviews of OHRQoL measures, studies reporting OHRQoL assessment through instruments, construction (development) and validation of a novel instrument, questionnaires that had a single item, and translation into Portuguese from Portugal were excluded.

### Search strategy

The studies were obtained through electronic searches in the United States National Library of Medicine (PubMed)/Medical Literature Analysis and Retrieval System Online (MEDLINE), Web of Science, Latin American and Caribbean Health Sciences Literature (Lilacs), Virtual Health Library (VHL)/(*Biblioteca Regional de Medicina* - BIREME), Scientific Electronic Library Online (SciELO), and Embase databases. The keywords used were searched in Health Sciences Descriptors (DeCS), Medical Subject Headings (MeSH), and published manuscripts on OHRQoL.

The Boolean operators AND and OR were used in combination with the following terms: quality of life, oral health quality of life, instrument, scale, questionnaire, measurement, measurement tool, psychometrics, reliability, validity, instrument validation, cross-cultural adaptation, instrument translation, Brazilian version, Brazil, Portuguese, and Brazilian Portuguese. A general search strategy was adapted to the characteristics of each database (Table 1) to identify studies of interest for this review. Databases were explored for articles and abstracts with no language restriction. In addition, a complementary scan on the gray literature through Google Scholar was performed. References in all included studies were checked for additional studies. The investigation in the respective databases were performed until December 2022.

**Table 1.** Search strategy utilized for each database.

Database	Search query
PubMed/MEDLINE LILACS VHL (BIREME)	(quality of life OR oral health quality of life OR instrument OR scale OR questionnaire OR measurement OR measurement tool) AND (psychometrics OR reliability OR validity) AND (instrument validation OR cross cultural adaptation OR instrument translation) AND (Brazilian version OR Brazil OR Portuguese OR Brazilian Portuguese)
Web of Science	#1: TS=(quality of life OR oral health quality of life OR instrument OR scale OR questionnaire OR measurement OR measurement tool) #2: TS=(psychometrics OR reliability OR validity) #3: TS=(instrument validation OR cross-cultural adaptation OR instrument translation) #4: TS=(Brazilian version OR Brazil OR Portuguese OR Brazilian Portuguese) #5: #1 AND #2 AND #3 AND #4
SciELO	(quality of life OR oral health quality of life OR instrument OR scale OR questionnaire OR measurement OR measurement tool) AND (psychometrics OR reliability OR validity)
Embase	#1: (quality of life OR oral health quality of life OR instrument OR scale OR questionnaire OR measurement OR measurement tool) #2: (psychometrics OR reliability OR validity) #3: (instrument validation OR cross-cultural adaptation OR instrument translation) #4: (Brazilian version OR Brazil OR Portuguese OR Brazilian Portuguese) #5: #1 AND #2 AND #3 AND #4

TS: topics.

### Study selection

The Rayyan tool (https://rayyan.qcri.org/welcome) was used in the selection of studies, management, and citation of references during the development of this review^11^. The selection process was performed by three reviewers (DWDO, FSL, and YGG) in two stages. In the first phase, the they independently identified all relevant studies through electronic search methods based on the eligibility criteria applied to titles and abstracts. For studies that seemed to meet the inclusion criteria or for which sufficient data were not found in the title and abstract to make a clear decision, the complete text was pre-selected. In the second phase, the pre-selected studies were read in full by the same researchers to decide whether or not the it met the inclusion criteria. When necessary, the authors of the papers were contacted by email to clarify questions related to the research. All the studies excluded, at this point or later, were recorded along with the reasons for rejection. Observational studies that met the eligibility criteria were included in the final analysis and submitted to data synthesis. Articles found twice or more were considered only once. Disagreements were resolved by consensus among the three reviewers; this procedure was applied at all stages. The reviewers were trained for each database before the research.

### Data extraction

Data were recorded qualitatively to allow comparisons among the selected studies, and each researcher qualitatively assessed them through an evaluation form. Data were collected on the following items: author, year of publication, country, study design, characteristics of the participants (sex and mean age), original language of the instrument, cross-cultural adaptation process, target population, main reported results, conclusion, name of the instrument, acronym, generality or specificity of the instrument, method of completion, domains, number of items, scoring, assessment period, time of completion, availability of the questionnaire in Brazilian Portuguese, internal consistency, criterion validity, construct validity, reliability, discriminant validity, translation, back-translation, synthesis, committee approach, pre-test, and psychometric evaluation.

### Assessment quality

The included studies had their quality assessed by the psychometric validation and adaptation process^10^. The identified psychometric properties were then evaluated according to nine assessment criteria: content validity, internal consistency, criterion validity, construct validity, reproducibility, responsiveness, floor and ceiling effects, and interpretability. It was assigned a positive (+), undetermined (?), or negative (-) rating for each of these measures, or zero (0) if no information was available. It was recommended to present the evaluation results in a table but not using an overall score, as this gives equal importance to each psychometric property^12^.

The cross-cultural adaptation was evaluated according to established guidelines^13^, namely:

1. Translation;
2. Back-translation;
3. Committee review;
4. Pre-test; and
5. Re-examination of score weighting.

In the first step, at least two qualified translators translated the scale from the original language into the target language. In the second step, two independent translators translated the translated version back into the original language, in order to ensure that it reflected the content of the original version. The third step ideally involved a committee review to develop the next-to-last version for pre-testing, and the fourth step consisted of applying this version to 30-40 individuals from the target population. The final step aimed to re-examine the score weighting, considering the cultural context. It was assigned present, absent, or unclear for each item.

### Certainty assessment

The certainty of the evidence was assessed according to COSMIN guidelines^10^, using the GRADEpro program, depending on each factor analyzed (risk of bias, indirect evidence, inconsistency, and imprecision), being classified as high, moderate, low, or very low. It started with high quality and was reduced by one or two levels when serious or very serious risk of bias, indirect evidence, inconsistency, or imprecision were identified. The outcomes assessed were psychometric analysis and cross-cultural adaptation.

## RESULTS

### Search and selection

**Figure 1.**
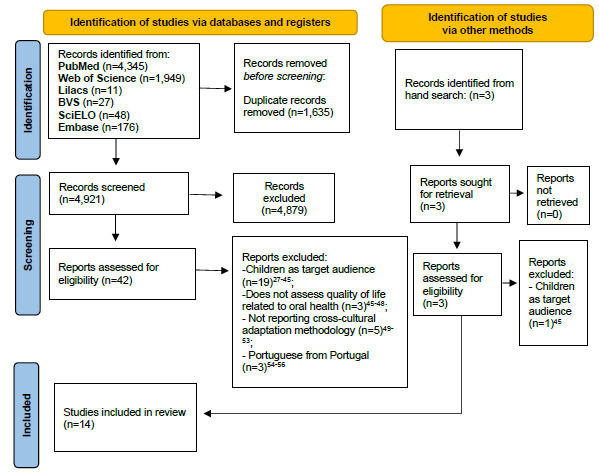
Flowchart of the included studies.

The electronic search retrieved 6,556 articles, of which 1,635 duplicates were removed. The manual search identified three additional studies. In the first phase, 4,879 publications were excluded. In the second, 30 studies were excluded. Therefore, 14 articles^3^ ^,^ ^14^ ^,^ ^15^ ^,^ ^16^ ^,^ ^17^ ^,^ ^18^ ^,^ ^19^ ^,^ ^20^ ^,^ ^21^ ^,^ ^22^ ^,^ ^23^ ^,^ ^24^ ^,^ ^25^ ^,^ ^26^ were considered in this review (Figure 1)^27^ ^,^ ^28^ ^,^ ^29^ ^,^ ^30^ ^,^ ^31^ ^,^ ^32^ ^,^ ^33^ ^,^ ^34^ ^,^ ^35^ ^,^ ^36^ ^,^ ^37^ ^,^ ^38^ ^,^ ^39^ ^,^ ^40^ ^,^ ^41^ ^,^ ^42^ ^,^ ^43^ ^,^ ^44^ ^,^ ^45^ ^,^ ^46^ ^,^ ^47^ ^,^ ^48^ ^,^ ^49^ ^,^ ^50^ ^,^ ^51^ ^,^ ^52^ ^,^ ^53^ ^,^ ^54^ ^,^ ^55^ ^,^ ^56^.

### Qualitative assessment

All revised studies^3^ ^,^ ^14^ ^,^ ^15^ ^,^ ^16^ ^,^ ^17^ ^,^ ^18^ ^,^ ^19^ ^,^ ^20^ ^,^ ^21^ ^,^ ^22^ ^,^ ^23^ ^,^ ^24^ ^,^ ^25^ ^,^ ^26^ presented a cross-sectional design and were carried out in Brazil. The number of participants ranged from 12^14^ ^,^ ^17^ to 504^24^. The age of the participants ranged from 24^21^ ^,^ ^24^ ^,^ ^25^ to 69 years^23^. Two studies^18^ ^,^ ^23^ did not undergo the cross-cultural adaptation process (supplementary material 1).

Supplementary material 2 shows the health conditions evaluated by the instruments. The self-completion method and the interview were used to fill out the questionnaires^3^ ^,^ ^14^ ^,^ ^15^ ^,^ ^16^ ^,^ ^17^ ^,^ ^18^ ^,^ ^19^ ^,^ ^20^ ^,^ ^21^ ^,^ ^22^ ^,^ ^23^ ^,^ ^24^ ^,^ ^25^ ^,^ ^26^. Four studies did not report the scoring^16^ ^,^ ^17^ ^,^ ^21^ ^,^ ^23^. The adapted instrument was available in six publications^3^ ^,^ ^14^ ^,^ ^15^ ^,^ ^17^ ^,^ ^23^ ^,^ ^24^.

Two studies^14^ ^,^ ^17^ did not report psychometric validation (Table 2). All adaptation steps were disclosed in 11 studies^3^ ^,^ ^14^ ^,^ ^15^ ^,^ ^16^ ^,^ ^17^ ^,^ ^19^ ^,^ ^20^ ^,^ ^21^ ^,^ ^24^ ^,^ ^25^ ^,^ ^26^ (Table 3).

**Table 2.** Psychometric assessment of oral health-related quality of life instruments.

Study	Content validity	Internal consistency	Criterion validity	Construct validity	Reliability	Discriminant validity	Cronbach’s global alpha value	Overall ICC value
Hanan et al.,^25^	+	+	+	+	+	+	0.92	0.92-0.97
Perazzo et al.,^26^	-	+	-	+	+	+	>0.80	0.84
Almeida et al.,^14^	+	-	-	-	-	-	NR	NR
Silveira et al.,^15^	+	+	+	+	+	-	0.96	0.93
Douglas-De-Oliveira et al.,^3^	+	+	+	+	+	+	0.95	0.96
Abegg et al.,^16^	+	+	+	+	+	+	0.69	0.69
Araújo et al.,^17^	+	-	-	-	-	-	NR	NR
Gava et al.,^18^	-	+	+	+	+	+	0.95	0.90
Kallás et al.,^19^	+	+	+	+	+	-	0.91-0.92	0.94
Campos et al.,^20^	+	+	+	+	+	+	0.87-0.91	0.82-0.89
Sardenberg et al.,^21^	+	+	+	+	+	+	0.75-0.91	0.89-0.99
Bortoluzzi et al.,^22^	+	+	+	+	+	-	0.78-0.89	0.78-0.89
Souza et al.,^23^	+	+	-	-	+	-	0.86	0.57
Oliveira et al.,^24^	+	+	+	+	+	+	0.91	0.87

ICC: intraclass correlation coefficient; NR: not reported.

**Table 3.** Cross-cultural adaptation assessment of oral health-related quality of life instruments.

Study	Translation	Back translation	Synthesis	Committee’s approach	Pre-test	Psychometric evaluation
Hanan et al.,^25^	Present	Present	Present	Present	Present	Present
Perazzo et al.,^26^	Present	Present	Present	Present	Present	Present
Almeida et al.,^14^	Present	Present	Present	Present	Present	Absent
Silveira et al.,^15^	Present	Present	Present	Present	Present	Present
Douglas-De-Oliveira et al.,^3^	Present	Present	Present	Present	Present	Present
Abegg et al.,^16^	Present	Present	Present	Present	Present	Present
Araújo et al.,^17^	Present	Present	Present	Present	Present	Absent
Gava et al.,^18^	?	?	?	?	?	Present
Kallás et al.,^19^	Present	Present	Present	Present	Present	Present
Campos et al.,^20^	Present	Present	Present	Present	Present	Present
Sardenberg et al.,^21^	Present	Present	Present	Present	Present	Present
Bortoluzzi et al.,^22^	?	?	?	?	?	Present
Souza et al.,^23^	Present	Present	Absent	Absent	Absent	Present
Oliveira et al.,^24^	Present	Present	Present	Present	Present	Present

### Certainty assessment

The certainty of the evidence was downgraded by the risk of bias and indirectness, being considered low for psychometric analysis outcome and moderate for cross-cultural adaptation outcome (Table 4).

**Table 4.** Level assessment of systematic reviews.

Certainty assessment							Certainty
Number of studies	Study design	Risk of bias	Inconsistency	Indirectness	Imprecision	Other considerations	
Psychometric analysis							
14	Observational studies	Serious*	Not serious	Serious*^,†^	Not serious	Very strong association	⨁⨁◯◯ Low
Cross-cultural adaptation							
14	Observational studies	Not serious	Not serious	Serious^†^	Not serious	Very strong association	⨁⨁⨁◯ Moderate

*studies that did not perform psychometric analysis; ^†^generic and specific instruments evaluated.

## DISCUSSION

The assessment of individuals’ oral health based only on clinical criteria makes it difficult to identify and recognize its impact on general well-being and people’s lives^4^. Due to this multidimensionality, the use of instruments is necessary to properly assess the patients, recording their subjectivity in a standardized and reproducible way^3^ ^,^ ^26^. In the present review, 14 OHRQoL instruments were adapted to Brazil and psychometrics properties were evaluated. All of them proved to be valid and ready for use.

The included instruments had the original language in English. To understand the OHRQoL in Portuguese-speaking population, the scales had to be properly translated and culturally adapted for use in this population^57^. All the studies were carried out in Brazil, which was expected, as the they adapted instruments to be used in the Brazilian culture. The studies were designed in accordance with Beaton et al.^6^, who recommended a cross-sectional study, indicating that data were collected at a specific time without additional follow-up. Researchers who carry out cross-sectional analyses generally have greater difficulty creating a consistent report on interventions, treatments, and other variables in quality of life^5^.

All instruments used to assess quality of life must have the basic properties of reproducibility, validity, and sensitivity to changes^58^. In this review, both specific^3^ ^,^ ^14^ ^,^ ^15^ ^,^ ^17^ ^,^ ^18^ ^,^ ^19^ ^,^ ^20^ ^,^ ^21^ ^,^ ^22^ ^,^ ^23^ ^,^ ^25^ and generic^16^ ^,^ ^24^ ^,^ ^26^ instruments were found, indicating that Brazilian researchers can use different instruments according to the research intention and/or oral condition. When specific instruments are not available for a particular condition, generic instruments are used, developed to reflect the impact of general oral health on the individuals` life. One of the main limitations of their use is the inability to detect small differences after intervention or in a specific condition of low reproducibility^59^. On the other hand, specific instruments individually assess the impact of a given condition and have greater sensitivity and detection capacity to some type of change in the study after a determined intervention^58^ ^,^ ^60^.

The notoriety of cross-cultural adaptation lies in producing instruments that are equivalent in different cultures, maintaining their content and validity in a different cultural context^61^. Through these instruments, it is possible to enable a better form of expression, language understanding and evaluation, allowing the best results of an investigation, and consequently, the promotion of care humanization^62^). Most of the reviewed studies followed the adaptation guidelines satisfactorily. It is estimated that the Brazilian culture experiences and the country’s context were inserted in the validated instruments that were prepared to be used in Brazil.

However, some studies have shown to be deficient in the cross-cultural adaptation process^18^ ^,^ ^22^ ^,^ ^23^. A flawed translation and adaptation process will affect the instrument’s reliability, creating an inconsistency between the original and the translated version, which may compromise the validity and reliability of an item and/or the domain of the instrument^6^ ^,^ ^57^. These psychometric properties express the information about the instruments validity, helping the researcher choose the potential instrument.

Two studies in this review^14^ ^,^ ^17^ did not assess the psychometric criteria suggested by Terwee et al.^12^. Through psychometric tests, it is possible to verify the instrument’s reliability and whether it measures what it is intended. After these psychometric tests are applied, a report is sent to the reader regarding the instrument’s reliability and validity. Consequently, when a researcher intends to investigate a specific or generic oral condition, these instruments become effective since they had undergone the whole psychometrics validation. The advantage of using adapted and validated instruments is time and effort savings, in addition to avoiding erroneous comparisons between different translated versions^6^.

A traditional method to estimate the reliability of the internal consistency of a questionnaire is Cronbach’s alpha^63^. It measures the correlation between answers by analyzing the profile of the responses given by the participants^64^ ^,^ ^65^. The minimum acceptable value for alpha is 0.70; on the other hand, very high values (greater than 0.90) may be related to redundancy or duplication of items, which may mean that several items measure the same construct^66^. In this case, duplication or redundancy must be eliminated. The studies used in this review had an alpha ranging from 0.69^16^ to 0.96^17^, demonstrating that the instruments adapted for Brazil have an ideal coefficient, reinforcing their reliability.

The included studies^3^ ^,^ ^14^ ^,^ ^15^ ^,^ ^16^ ^,^ ^17^ ^,^ ^18^ ^,^ ^19^ ^,^ ^20^ ^,^ ^21^ ^,^ ^22^ ^,^ ^23^ ^,^ ^24^ ^,^ ^25^ ^,^ ^26^ presented two methods for recording the individuals’ reports: the self-completion method (self-report scale), in which the instrument is filled out by the participant, requiring greater individual cooperation^67^; and the interview in which the instrument is completed by the observer, which may present problems due to the interference of the interviewer’s experience^64^ ^,^ ^68^. Researchers need to pay attention to this fact when applying the instrument to their research or clinical activity in order to prevent information bias^69^.

The time to fill the instrument was not reported in most studies. This is significant data to be gathered, as the researcher should be aware of the time that will be allocated to the data collection when using the instrument.

The indication of the period to be considered in the participant’s response was not reported in most of the studies as well^16^ ^,^ ^18^ ^,^ ^19^ ^,^ ^20^ ^,^ ^21^ ^,^ ^22^ ^,^ ^23^ ^,^ ^24^ ^,^ ^25^ ^,^ ^26^. This information is important because the Brazilian version will be applied and reapplied frequently and, as the author did not include the evaluation period, there may be a response^70^ and/or methodological^71^ bias, compromising the investigation.

The ultimate objective of cross-cultural adaptation is to produce an instrument to be applied to a population with culture and/or language different from the original instrument^6^ ^,^ ^28^. However, some reviewed studies did not provide a Brazilian version of the adapted instruments^16^ ^,^ ^18^ ^,^ ^19^ ^,^ ^20^ ^,^ ^21^ ^,^ ^22^ ^,^ ^25^ ^,^ ^26^. This can limit their use and citation, or even stimulate other authors to develop a similar instrument.

The GRADEpro tool is widely used to verify the quality and certainty of the evidence in systematic reviews^72^. The overall certainty of the evidence in this review can be described as moderate to low, indicating significant constraints on the imprecision of results or lack of data to support a strong conclusion. As a result, recommendations based on this evidence are less secure and subject to change as new information emerges^73^. It is important to note that a moderate to low certainty rating does not necessarily imply that the instruments in question are ineffective or harmful. This simply indicates that the available evidence is not robust enough to provide a definitive answer^74^, indicating the need for caution in clinical decision-making.

The assessment of the quality of life includes subjective experiences that contribute to an individual’s evaluation parameter^75^. The PROMs are questionnaires that collect health outcomes directly from the people who experience them^76^. In addition, these instruments allow the investigation of symptoms, quality of life, functional and emotional status, and dysfunctions, as well as contribute to the decision, planning, and evaluation of certain types of treatment^77^. PROMs can be seen as important strategies to support clinical decisions and most indicated treatments, compare the results among health professionals, encourage quality improvement, and evaluate public health practices and policies^78^. Thus, measuring the disease’s impact on the patient’s quality of life becomes an increasingly essential tool, especially when its properties have been reaffirmed as valid and reproducible parameters. This review showed that several instruments, specific or generic, are available with the purpose of assessing the OHRQoL of Brazilian adult patients.

The present review found some limitations in the included studies, such as the lack of information in some reviewed studies, non-availability of Brazilian instruments, and absence of longitudinal validation of the reviewed instruments. It is suggested that cross-sectional studies be carried out to longitudinally validate the OHRQoL instruments adapted for Brazil. Researchers are encouraged to publish their validated OHRQoL instruments.

In conclusion, there are 14 OHQoL instruments adapted for Brazilian adults. The major studies provided information regarding the exact processes of validation, translation, and cultural adaptation. Additionally, three papers failed to prove the validity of quality of life among oral potentially malignant disorder (OPMD QoL), orthognathic quality of life questionnaire (OQLQ), and oral health impact profile-edent (OHIPEDENT) instruments, making it difficult for researchers to choose based on psychometric properties. In general, the generic and specific OHRQoL instruments adapted for the Brazilian adult population can be used with caution by researchers and clinical dentists in Brazil, since they presented moderate to low certainty of the evidence. Specifically, the OPMD QoL, OQLQ, and OHIPEDENT scales require further validation.
