# Supplementary material for: Psychometric assessment of oral health-related quality of life questionnaires cross-culturally adapted for use in Brazilian adults - a systematic review
Source: Rev Bras Epidemiol. 2023 Oct 27;26:e230046. doi: 10.1590/1980-549720230046 (PMC10626907; doi:10.1590/1980-549720230046)
Supplement: Supplementary file 1 [file 1980-5497-rbepid-26-e230046-s1.pdf]

Table 2. Characteristics of the reviewed studies.

Study	Country	Study design	Participants	Original language	Cross-cultural adaptation	Psychometric validation	Target population	Main results reported	Conclusion
**Hanan et al., 2022**	Brazil	Cross-sectional	29 men and 50 women, ±24.4 years old.	English	Present	Present	Adults	Both stability and internal consistency were adequate. A positive correlation between OHIP-Aes-Braz and OHIP-14 (r s =0.63) and OIDP (r s =0.77) was observed.	The OHIP-Aes-Braz showed good psychometric properties and showed sensitivity to changes in esthetic evaluation in Brazilian adults submitted to tooth bleaching.
**Perazzo et al., 2022**	Brazil	Cross-sectional	62 men and 147 women, ± 39.36 years old.	English	Present	Present	Adults	The B-POHW demonstrated satisfactory internal consistency (Cronbach's α and McDonald's ω > 0.8); the intraclass correlation coefficient suggested good reliability for the Global Factor of B-POHW (ICC = 0.84); evidence based on other variables and construct representation	The B-POHW is psychometrically sound to be used in a Brazilian context, and evidence of its internal structure confirmed its theoretical framework fot measuring positive oral health.

								was in line with the positive oral health framework.	
**De Almeida et al., 2021**	Brazil	Cross-sectional	12 participants, ±61.5 years old.	English	Present	Absent	Adults with potentially malignant oral disorders	The content validation, pre-test and operational equivalence indicators indicate that the Brazilian version of this questionnaire is a promising instrument.	A tool that seems to be valid for assessing the quality of life of people with potentially malignant oral diseases.
**Silveira et al., 2020**	Brazil	Cross-sectional	83 men and 117 women, ±41.34 years old.	English	Present	Present	Adults	Factor analyses confirmed the unidimensionality of the B-HALT, which also demonstrated excellent internal consistency and test-retest reliability. There was a positive correlation between B-HALT and organoleptic scores ($r = 0.33$; $p < 0.001$). B-HALT was able to outline the groups with and without halitosis.	The B-HALT proved to be a reliable and valid tool to assess halitosis-associated health-related quality of life in Brazilian adults.
**Douglas de Oliveira et al., 2018**	Brazil	Cross-sectional	69 men and 131 women, 30.4 years of age	English	Present	Present	Adults with dentine hypersensitivity.	Cronbach's $\alpha = 0.945$; intraclass correlation coefficient = 0.959, $p < 0.001$; a significant correlation between the	This study provides evidence supporting the cross-cultural validity of the

								DHEQ-15 domains and the global assessment of oral health (p<0.001). For criterion and construct validity, there was an inverse correlation between self-rated oral health, perceived oral care needs and satisfaction with oral health, and OIDP score. Cronbach's α coefficient ranged from 0.69 to 0.67. The test-retest reliability was 0.69 (ICC).	Brazilian version of the DHEQ-15 for use in Brazil.
**Abegg et al., 2015**	Brazil	Cross-sectional	117 men and 83 women, 60.2 years old.	English	Present	Present	Adults	Cronbach's α = 0.945; intraclass correlation coefficient = 0.959, p < 0.001; a significant correlation between the DHEQ-15 domains and the global assessment of oral health (p<0.001). For criterion and construct validity, there was an inverse correlation between self-rated oral health, perceived oral care needs and satisfaction with oral health, and OIDP score.	The validation process showed that the Brazilian OIDP has the necessary basic psychometric properties to be used in the 50-74 age group in Brazil.

								Cronbach's α coefficient ranged from 0.69 to 0.67. The test-retest reliability was 0.69 (ICC).	
**De Araújo et al., 2013**	Brazil	Cross-sectional study	9 men and 3 women, ±25 years old.	English	Present	Absent	Adults undergoing orthosurgical treatment	The OQLQ was translated into Brazilian Portuguese. This translation was tested in a pilot study with 12 patients, between 16 and 34 years old.	The translation of the OQLQ into Brazilian Portuguese proved to be an adequate tool to access the impact of dentofacial deformities on the quality of life of patients in the Brazilian public health system who need orthosurgical treatment.
**Gava et al., 2013**	Brazil	Cross-sectional	59 men and 42 women, ±26.5 years old.	English	Absent	Present	Adults	Cronbach's α and ICC were 0.95 and 0.90. The B-OQLQ domains that had the greatest effect on the quality of life included "social aspects of deformity" and "facial esthetic".	The Brazilian version of the OQLQ proved to be valid and reliable, with good psychometric properties and can be considered an adequate tool to assess the effect of

									dentofacial deformities on the quality of life of individuals with this condition.
**Kallás et al., 2013**	Brazil	Cross-sectional	7 men and 43 women, ±40.7 years old.	English	Present	Present	Adults	Internal consistency (Cronbach's α = 0.9), interobserver (ICC = 0.92) and intraobserver (ICC = 0.98) correlations showed high scores. The validity of Brazil-MOPDS in relation to OHIP-14 (r = 0.85) and VAS (r = 0.75) showed high correlations.	The Brazil-MOPDS was successfully translated and adapted to be applied to Brazilian patients, with satisfactory internal and external reliability.
**Campos et al., 2012**	Brazil	Cross-sectional	115 men and 134 women, ± 36.84 years old.	English	Present	Present	Adults	The reproducibility was excellent in both dimensions 'functional capacity' and 'feeding'. Items 1, 2, 6 and 7 of D1 had factor weights below the desired cutoff point. Convergent validity was adequate for both factors. However, its discriminant validity was low. Internal consistency was excellent.	The Portuguese version of the reduced MFIQ produced data with good validity and reliability.

**Sardenberg et al., 2011**	Brazil	Cross-sectional	124 men and 121 women, ±24 years old.	English	Present	Present	Adults with malocclusion (particularly in the anterior region)	The Cronbach's α of the subscales was between 0.75 and 0.91 and the test-retest reliability ranged from 0.89 to 0.99. Discriminant validity revealed that individuals without malocclusion had different PIDAQ scores compared to those with malocclusion.	The results suggest that the Brazilian version of the PIDAQ has satisfactory psychometric properties and, therefore, applies to young adults in Brazil.
**Bortoluzzi et al., 2011**	Brazil	Cross-sectional	9 men and 16 women, ±25.4 years old.	English	Present	Present	Adults with dentofacial deformity or orthognathic treatment.	The internal consistency analysis of the OQLQ-Brazil showed a good correlation for items or domains, and the test-retest reliability also showed excellent intraclass correlation coefficients. The Brazilian-OQLQ showed a weak and negative correlation with the SF-36 and a good correlation with the OHIP-49.	The demonstration of its reproducibility, reliability and makes this instrument another useful parameter for assessing the impact of dentofacial deformity on the quality of life of Brazilians.

**Souza et al., 2007**	Brazil	Cross-sectional	23 men and 42 women, ±69.1 years old.	English	Absent	Present	Adults/Elderly	Internal consistency results showed a Cronbach's α of 0.90. Through test-retest analysis, an intraclass correlation coefficient of 0.57 was found, and individual responses reflected a wide range of agreement. Interviewed volunteers understood most of the questions well.	The Brazilian version of the OHIP-EDENT is suitable for assessing the oral health-related quality of life of edentulous individuals.
**De Oliveira et al., 2005**	Brazil	Cross-sectional	504 women, ±24 years old.	English	Present	Present	Adults	Test-retest stability and internal consistency were adequate. The construct validity was confirmed by the correlation between the OHIP14 scores with self-perception of general and oral health, and by the differences in the scores of the groups formed.	The Brazilian version of the OHIP14 has good psychometric properties, similar to those of the original instrument.

±: mean age

Table 3. Characteristics of oral health-related quality of life instruments.

Study	Instrument	Abbreviation	Generic/specific instrument	Completion method	Domain names	Number of items	Score	Evaluation period	Completion time	Available instrument
**Hanan et al., 2022**	Oral Health Impact Profile - Aesthetic Questionnaire	OHIP-Aes-Braz	Specific condition: aesthetic dental treatment	Self-completion	Physiological discomfort, pain, physiological disability, physical disability, functional limitation, disability, and social disability.	14	The response scale is a 4-point Likert scale. A total score is calculated by summing up the item scores, ranging 0 to 56. A higher score indicates a worse aesthetic impact on quality of life.	30 days	NR	No
**Perazzo et al., 2022**	Positive Oral Health and Well-Being	B-POHW	Generic Instrument	Self-completion	subjective–psychological, functional–social, and biological-physical	15	The response scale is a 4 point Likert scale. A total score is calculated by summing up the item	NR	NR	No

							scores. Higher scores indicate greater positive health.			
**De Almeida et al., 2021**	Quality-of-life questionnaire for patients with oral potentially malignant lesions	OPMD QoL	Specific condition: patients with oral potentially malignant lesions	Interview	Difficulties in diagnosis, physical disability and limitations, psychological and social well-being, effects of treatment on daily activities.	20	The summary score of the questionnaire ranges from 0 to 80, with a higher score indicating a lower quality of life.	NR	10 minutes	Yes
**Silveira et al., 2020**	Halitosis Associated Life-Quality	HALT	Specific condition: Halitosis	Self-completion	Oral symptoms, social well-being, emotional well-being.	20	The final score ranging from 0 to 100. Higher scores indicate a greater impact of halitosis in OHRQoL	NR	10 minutes	Yes
**Douglas de Oliveira et al., 2018**	Dentine Hypersensitivity Experience Questionnaire	DHEQ-15	Specific condition: individuals presenting dentin hypersensitivity	Self-completion	Restrictions, adaptation, social impact, emotional impact, and identity.	15	The total and subscale scores are calculated as the sum of all items. Higher DHEQ-15 scores	30 days	5 minutes	Yes

							indicate a negative impact on quality of life			
**Abegg et al., 2015**	Oral impact on daily performance	OIDP	Generic instrument	Interview	NR	12	NR	NR	NR	No
**De Araújo et al., 2013**	Orthognathic Quality of Life Questionnaire	OQLQ	Specific condition: patients in need of orthosurgical treatment	Interview	Social aspects of the deformity, facial aesthetics, oral functions, and awareness of the facial deformity.	22	NR	NR	15 to 25 minutes.	Yes
**Gava et al., 2013**	Orthognathic Quality of Life Questionnaire	B-OQLQ	Specific condition: patients with dentofacial alterations	Self-completion	Perceived oral condition, global assessment of the quality of life, acceptance of facial appearance.	22	The score for each individual can range from 0 to 88 points. A higher score represents a greater effect of problems regarding daily life and a decrease in quality of life	NR	NR	No
**Kallás et al., 2013**	Manchester Orofacial Pain Disability Scale	MOPDS	Specific condition: with	Interview	Physiological discomfort, pain, physiological	24	Score ranging from 0 (least impact on oral	NR	NR	No

			orofacial pain symptoms.		disability, physical disability, functional limitation, disability, and social disability.		health in the daily report) to 28 (most impact)			
**Campos et al., 2012**	Mandibular Function Impairment Questionnaire	MFIQ	Specific condition: patients with TMD	Self-completion	Functional Capacity, Feeding	17	The average of points attributed to each question allows the classification of individuals according to TMD severity.	NR	NR	No
**Sardenberg et al., 2011**	Psychosocial Impact of Dental Aesthetics Questionnaire	PIDAQ	Specific condition - assessing the psychosocial impact of dental aesthetics in young adults aged 18 to 30 years.	Self-completion	Aesthetic concerns, psychological impact, social impact, dental self-confidence.	23	NR	NR	NR	No
**Bortoluzzi et al., 2011**	Orthognathic Quality of Life Questionnaire	OQLQ	Specific condition - patients in need	Self-completion	Social aspects of deformity, facial aesthetics, oral functions, and	22	OQLQ scoring is performed by adding individual	NR	NR	No

			of orthosurgical treatment.		awareness of facial deformity.		items within domains. A total OQLQ score can range from 0 to 88, a higher score indicates a worse QoL			
**Souza et al., 2007**	Oral Health Impact Profile for assessing edentulous	OHIP-EDENT	Specific condition: edentulism.	Interview	Functional limitation; physical pain; psychological discomfort; physical disability; psychological disability; social disability; deficiency.	19	NR	3 months	NR	Yes
**De Oliveira et al., 2005**	Oral Health Impact Profile	OHIP14	Generic instrument	Interview	Functional limitation, physical pain, psychological discomfort, physical disability, psychological , social , and disability	14	The OHIP14 scale ranges from 0 to 56, with higher scores indicating worse OHRQoL.	6 months	NR	Yes

NR: not reported
